# PReS-FINAL-2180: Efficacy and safety of tocilizumab (TCZ) in patients with polyarticular-course juvenile idiopathic arthritis (pcJIA): 2-year data from CHERISH

**DOI:** 10.1186/1546-0096-11-S2-O15

**Published:** 2013-12-05

**Authors:** F De Benedetti, N Ruperto, Z Zuber, R Cuttica, R Xavier, I Calvo, N Rubio, E Alekseeva, V Chasnyk, J Chavez, G Horneff, V Opoka-Winiarska, P Quartier, A Spindler, C Keane, K Bharucha, J Wang, D Lovell, A Martini, HI Brunner

**Affiliations:** 1 IRCCS Ospedale Ped Bambino Gesú, Rome, Italy; 2 PRINTO, Genoa, Italy; 3 Pediatric Rheumatology Unit, St. Louis Children's Hospital, Krakow, Poland; 4 Roche, Welwyn, UK; 5 Genentech, San Francisco, CA, USA; 6 PRCSG, Cincinnati, OH, USA

## Introduction

Efficacy and safety of TCZ, an IL-6 receptor inhibitor, were previously demonstrated at wk 40 of CHERISH, a phase 3 trial in patients (pts) with pcJIA [1].

## Objectives

To investigate efficacy and safety of TCZ over 104 wks of treatment in pcJIA.

## Methods

Pts 2-17 years old with ≥6 months' active pcJIA who failed methotrexate received open-label (OL) TCZ (weight ≥30 kg, 8 mg/kg [n = 119]; weight <30 kg, randomised [1:1] to 8 [n = 34] or 10 [n = 35] mg/kg) every 4 wks for 16 wks. Pts with ≥JIA ACR30 response at wk 16 entered a 24-wk double-blind withdrawal period and were randomised (1:1) to placebo or continuation with TCZ. Pts with JIA ACR30 flare or who completed the withdrawal period entered an OL extension through wk 104.

## Results

188 pts entered the lead-in period, 166 entered the withdrawal period, 160 entered the OL extension period and 155 completed 104 wks. In pts who received TCZ throughout the study, JIA ACR responses and improvement in JIA ACR core components (Table) were maintained through wk 104. The safety population comprised 188 pts with 307 pt years (PY). Rates/100PY of AEs and serious AEs (SAEs) were 406.5 and 11.1, respectively; infections were the most common AE (151.4) and SAE (5.2). ALT and AST elevations ≥3 × upper limit of normal occurred in 6.4% and 2.7% of pts, respectively. Grade 3 neutropenia and grade 2/3/4 thrombocytopenia occurred in 5.9% and 1.6% of pts, respectively. LDL cholesterol ≥110 mg/dl occurred in 16.2% of pts.

**Table 1:** JIA ACR50/70 responses and percentage change from baseline in components,^a ^mean ± SD

	All TCZ (N = 82)	
	
	Wk 40	Wk 104
JIA ACR70 responders,^a ^n (%)	65 (79.3)	71 (86.6
JIA ACR90 responders,^a ^n (%)	41 (50.0)	58 (70.7)
Active joints (0-71)	-82.4 ± 24.9	-87.7 ± 27.1
Joints with limitation in ROM (0-67)	-73.5 ± 30.7	81.3 ± 31.7
Patient global^c ^(VAS 0-100 mm)	-62.5 ± 76.3	-75.4 ± 43.8
Physician global (VAS 0-100 mm)	-85.3 ± 16.8	-89.7 ± 23.7
CHAQ-DI (0-3)	-66.0 ± 44.7	-76.7 ± 34.7
ESR (mm/h)	-76.5 ± 22.0	-76.2 ± 27.3

^a^Pts who withdrew are excluded. ^b^Pts who withdrew due to non-safety reasons are non-responders. Pts who withdrew due to safety are included using last observation carried forward. ^c^Parent-rated.

## Conclusion

Efficacy of TCZ was maintained through 2 years of treatment in pts with pcJIA, with no change in safety profile from that reported previously [1].

## Disclosure of interest

F. De Benedetti Grant/Research Support from: Abbott, Pfizer, BMS, Roche, Novimmune, Novartis, S0BI, N. Ruperto Grant/Research Support from: Abbott, AstraZeneca, BMS, Centocor, Lilly, Francesco Angelini, GSK, Italfarmaco, Merck Serono, Novartis, Pfizer, Regeneron, Roche, Sanofi Aventis, Schwarz Biosciences, Xoma, Wyeth, Consultant for: Abbott, AstraZeneca, BMS, Centocor, Lilly, Francesco Angelini, GSK, Italfarmaco, Merck Serono, Novartis, Pfizer, Regeneron, Roche, Sanofi Aventis, Schwarz Biosciences, Xoma, Wyeth, Speakers Bureau: Abbott, Boehringer, BMS, Novartis, Astellas, Italfarmaco, MedImmune, Pfizer, Roche, Z. Zuber: None declared, R. Cuttica Speakers Bureau: Roche, Abbott, Pfizer, Novartis, BMS, R. Xavier: None declared, I. Calvo: None declared, N. Rubio: None declared, E. Alekseeva Grant/Research Support from: Roche, Abbott, Pfizer, BMS, Centocor, Novartis, Speakers Bureau: Roche, Merck, Abbott, BMS, Medac, Novartis, Pfizer, V. Chasnyk: None declared, J. Chavez: None declared, G. Horneff Grant/Research Support from: Abbott, Pfizer, V. Opoka-Winiarska: None declared, P. Quartier Grant/Research Support from: Abbott/Abbvie, Chugai-Roche, Novartis, Pfizer, Consultant for: Abbott/Abbvie, BMS, Chugai-Roche, Novartis, Pfizer, Servier, Sweedish Orphan Biovitrum, Speakers Bureau: BMS, Novartis, Pfizer, A. Spindler: None declared, C. Keane Employee of: Roche, K. Bharucha Employee of: Roche, J. Wang Employee of: Roche, D. Lovell Grant/Research Support from: NIH, Consultant for: AstraZeneca, Centocor, Janssen, Wyeth, Amgen, Bristol-Meyers Squibb, Abbott, Pfizer, Regeneron, Hoffmann-La Roche, Novartis, Genentech, Speakers Bureau: Genentech, Roche, A. Martini Grant/Research Support from: Abbott, AstraZeneca, BMS, Centocor, Lilly, Francesco Angelini, GSK, Italfarmaco, Merck Serono, Novartis, Pfizer, Regeneron, Roche, Sanofi Aventis, Schwarz Biosciences, Xoma, Wyeth, Consultant for: Abbott, AstraZeneca, BMS, Centocor, Lilly, Francesco Angelini, GSK, Italfarmaco, Merck Serono, Novartis, Pfizer, Regeneron, Roche, Sanofi Aventis, Schwarz Biosciences, Xoma, Wyeth, Speakers Bureau: Abbott, BMS, Astellas, Boehringer, Italfarmaco, MedImune, Novartis, Pfizer, H. Brunner Consultant for: Novartis, Genentech, MedImmune, EMD Serono, AMS, Pfizer, UCB, Janssen, Speakers Bureau: Genentech.
